# Sacubitril/Valsartan Improves Hemodynamic Parameters of Pulmonary and Systemic Circulation in Patients Awaiting Heart Transplantation

**DOI:** 10.3390/jcm14082539

**Published:** 2025-04-08

**Authors:** Arnold Péter Ráduly, Edward Saman Kothalawala, László Balogh, Zsuzsanna Majoros, Zsófia Pólik, László Fülöp, Ferenc Győry, László Nagy, Beáta Bódi, Máté Balázs Kovács, Zoltán Csanádi, Zoltán Papp, Balázs Muk, Attila Borbély

**Affiliations:** 1 Division of Cardiology, Department of Cardiology, Faculty of Medicine, University of Debrecen, 4032 Debrecen, Hungary; balogh.laszlo@med.unideb.hu (L.B.); fulop.laszlo@med.unideb.hu (L.F.); gyory.ferenc@med.unideb.hu (F.G.); dr.nagy.laszlo@med.unideb.hu (L.N.); kovacs.mate.balazs@med.unideb.hu (M.B.K.); csanadi.zoltan@med.unideb.hu (Z.C.); 2 Division of Clinical Physiology, Department of Cardiology, Faculty of Medicine, University of Debrecen, 4032 Debrecen, Hungary; zsofiapolik@med.unideb.hu (Z.P.); bodibea@med.unideb.hu (B.B.); pappz@med.unideb.hu (Z.P.); 3 Kálmán Laki Doctoral School, University of Debrecen, 4032 Debrecen, Hungary; 4 Department of Pediatrics, Faculty of Medicine, University of Debrecen, 4032 Debrecen, Hungary; kothalawala.edward@med.unideb.hu; 5 Cardiology Department, Central Hospital of Northern Pest-Military Hospital, 1134 Budapest, Hungary; zsmajoros@gmail.com; 6 Department of Adult Cardiology, Gottsegen National Cardiovascular Center, 1096 Budapest, Hungary; balazsmukmd@gmail.com

**Keywords:** advanced heart failure, right heart catheterization, heart transplantation, secondary pulmonary hypertension, sacubitril/valsartan

## Abstract

**Background/Objectives**: Heart transplantation (HTX) is the definitive treatment for advanced heart failure (AdHF). The angiotensin receptor neprilysin inhibitor (ARNI) sacubitril/valsartan (S/V) has been shown to reduce heart failure (HF) hospitalizations and mortality when compared to conventionally administered HF medications (i.e. angiotensin-converting enzyme inhibitors (ACEIs) and angiotensin II receptor blockers (ARBs)). Nevertheless, limited data are available on the hemodynamic (HD) effects of ARNI in patients with AdHF. Therefore, the aim of the present study was to compare echocardiographic, laboratory, and HD parameters relevant to HF before and after switching to ARNI in patients with AdHF awaiting HTX. **Methods**: A retrospective analysis was conducted utilizing available data on HD parameters, N-terminal pro-brain natriuretic peptide (NT-proBNP) levels, data on kidney function, HF therapy, and comorbidities. The study cohort comprised 13 AdHF patients (3 women, 10 men; mean age 56.4 ± 9 years) of whom 53.8% presented with non-ischemic and 46.2% with ischemic etiology. All patients were awaiting heart transplantation (HTX) and were transitioned to ARNI therapy between 2018 and 2021. **Results**: After switching to ARNI, we observed significant improvements: in left ventricular ejection fraction (LVEF: 27.27 ± 1.04% vs. 23.65 ± 1.02%, *p* = 0.03; data are given as mean ± SEM after vs. before ARNI therapy, respectively), cardiac output (CO: 4.90 ± 0.35 L/min vs. 3.83 ± 0.24 L/min, *p* = 0.013), and stroke volume (SV: 70.9 ± 5.9 mL vs. 55.5 ± 4.12 mL, *p* = 0.013). Significant reductions in systemic vascular resistance (SVR: 1188 ± 79.8 vs. 1600 ± 100 DS/cm^5^, *p* = 0.004) and pulmonary vascular resistance (PVR: 232.5 ± 34.8 vs. 278.9 ± 31.7 DS/cm^5^, *p* = 0.04) were also noted. Central venous pressure (CVP), pulmonary arterial systolic and diastolic pressures (PAPs and PAPd), pulmonary capillary wedge pressure (PCWP), and NT-proBNP levels did not exhibit significant changes upon ARNI administration. **Conclusions**: Early transition to ARNI therapy offers significant benefits for invasively measured hemodynamic parameters in patients with AdHF, potentially aiding in the stabilization and improvement of this vulnerable patient population.

## 1. Introduction

Heart failure (HF) with reduced ejection fraction (HFrEF) is a common condition in developed societies. This is largely due to increased cardiovascular risk factors, longer life expectancy, better survival rates after heart attacks, and improved HF management strategies [1,2]. Current HF guidelines recommend (1) renin angiotensin aldosterone system (RAAS) inhibitors (angiotensin-converting enzyme inhibitors (ACEIs) angiotensin II receptor blockers (ARBs) in cases of ACEI intolerance, or the angiotensin receptor neprilysin inhibitor (ARNI); (2) beta-blockers (BB); (3) mineralocorticoid receptor antagonists (MRA); and (4) sodium glucose cotransporter-2 (SGLT2) inhibitors (regardless of the presence or absence of diabetes) for all symptomatic HFrEF patients [3,4]. The ARNI offers several advantages over ACEI in HF management. The PARADIGM-HF trial demonstrated that ARNI is more effective than ACEI in reducing mortality and heart failure-related hospitalizations [5,6]. While the magnitude of the effects may vary, ARNI therapy generally leads to higher reductions in NT-proBNP levels—a marker of HF severity; it enhances hemodynamics, improves cardiac function by reducing afterload to a greater extent, increases natriuresis, and lowers systemic vascular resistance (SVR) more than those upon ACEI therapy. Patients on ARNI also experience improved functional status (NYHA class) and quality of life compared to those on ACEI [7,8]. Therefore, ARNI is now considered superior to ACEI in HFrEF management [4].

As a result of recent years’ effective drug and device therapies, survival of HF patients has improved significantly [9]. Nonetheless, despite maximally tolerated disease modifying therapy, progressive deterioration of cardiac pump function frequently occurs. Consequently, a significant proportion of HF patients enters the stage of advanced HF (AdHF), characterized by severe symptoms, frequent decompensations, and poor prognosis [10,11,12], whereby heart transplantation (HTX) is considered as a gold standard therapy for eligible patients. Surprisingly little data are available for the ARNI treatment evoked hemodynamic (HD) changes in patients with AdHF [13]. Therefore, the aim of this study was to investigate the effects of ARNI on invasive HD parameters in patients with AdHF awaiting HTX.

## 2. Materials and Methods

### 2.1. Study Population, Measured Parameters

We conducted a retrospective analysis of patients with AdHF who were undergoing pre-transplant evaluation or were already on the HTX waiting list and whose ACEI or ARB therapy was transitioned to ARNI between 2018 and 2021 in the Department of Cardiology and Cardiac Surgery at the University of Debrecen and in the Cardiology Department, Central Hospital of Northern Pest-Military Hospital, Budapest. Risk factors, comorbidities, laboratory parameters relevant for HF, and pharmacological and device therapy were analyzed. To assess the efficacy of ARNI treatment, changes in left ventricular ejection fraction (LVEF) and serum NT-proBNP levels were consecutively evaluated. LVEF was measured via transthoracic echocardiography using Simpson’s biplane method by means of a Philips EPIQ 7C Ultrasound system. Thirteen patients who were transitioned from ACE inhibitors or ARBs to ARNI and underwent Swan–Ganz (SG) catheterization both prior to and following the therapy switch were included in the analysis

### 2.2. Right Heart Catheterization

An ultrasound-guided jugular vein puncture was performed to insert a Swan–Ganz (SG) catheter after administering local anesthesia (1% lidocaine) under sterile conditions. Continuous multiparametric intensive monitoring, including non-invasive blood pressure, SpO_2_ measurement, and electrocardiography, was included throughout the procedure. After advancing the catheter through an 8F sheath, central venous pressure (CVP) and right atrial pressure were measured, followed by right ventricular pressure determination upon crossing the tricuspid valve. Pulmonary systolic and diastolic pressures were then measured, and pulmonary capillary wedge pressure (PCWP) was recorded at the end of expiration. Cardiac output (CO) was determined using the thermodilution method, involving cold saline injection into the proximal port of the SG catheter. Pulmonary vascular resistance (PVR), systemic vascular resistance (SVR), and stroke volume (SV) were subsequently calculated. In patients with post-capillary (secondary) pulmonary hypertension, reversibility was assessed by administering a continuous epoprostenol infusion, followed by repeated measurements.

### 2.3. Statistics

Statistical analysis was performed by using GraphPad Prism 9.0. For normally distributed data, paired *t*-tests were used, whereas Wilcoxon matched-pairs signed rank tests were applied for non-normally distributed data. Normality was assessed using the Shapiro–Wilk test. Data are given as the mean ± SEM or median and IQR. Statistical significance was defined as *p* < 0.05.

## 3. Results

### 3.1. Patient Characteristics

The study population had a male predominance (77% male; 23% female). About half of the patients had ischemic HF, while the other half had dilated cardiomyopathy. All patients had AdHF (NYHA class III-IV). Relevant comorbidities and risk factors for HF were also assessed; hypertension and atrial fibrillation were present in approximately half of the patients, and 31% had a history of type-2 diabetes mellitus and previous myocardial infarction (Table 1). Overweight and obesity were prevalent, affecting 50% and 30% of the patients, respectively, and 40% had a history of smoking. 

All patients received optimized medical and device therapy, including ACEIs or ARBs, BBs, MRAs, and ICD/CRT-D in accordance with the relevant guidelines (Table 1). At the time of the study, SGLT2 inhibitors were not included in the HF guidelines; they were given only to patients presenting with type-2 diabetes. Despite receiving the maximum tolerated doses of HF medications and adequate device therapy, patients remained symptomatic, prompting a switch from ACEI/ARB to ARNI. At the first follow-up visit (median time: 6.8 months), the target ARNI dose (97/103 mg twice daily) was achieved in three patients (23%). Among the remaining patients, seven (54%) attained a half-dose, while three (23%) reached a quarter-dose. The up-titration of the ARNI was opposed primarily by symptomatic hypotension.

### 3.2. Effectiveness and Safety of ARNI—Changes in NYHA Class, LVEF and NT-proBNP

**Figure 1:**
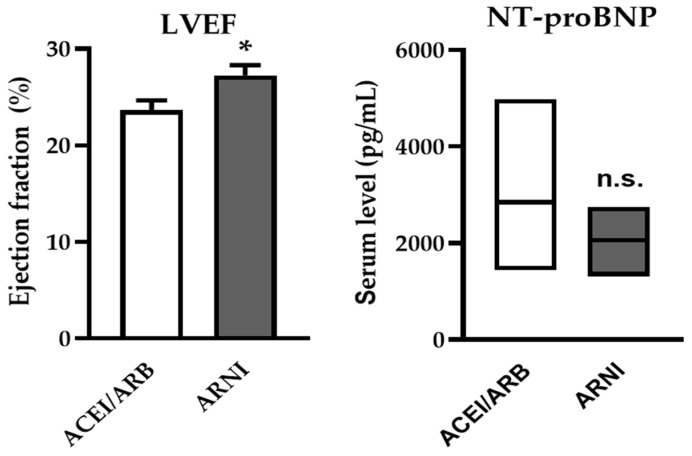
**Effects of ARNI on LVEF and NT-proBNP levels of AdHF patients awaiting HTX**. After switching to ARNI, left ventricular ejection fraction (LVEF) increased significantly. N-terminal pro-brain natriuretic peptide (NT-proBNP) levels did not change but showed a trend toward a decrease. (Data are given as mean ± SEM for LVEF, median and IQR for NT-proBNP (N = 13) * *p* < 0.05; n.s., non-significant).

After switching to ARNI, a significant increase in LVEF was observed at the first follow-up examination (27.27 ± 1.04% vs. 23.65 ± 1.02%, *p* = 0.016) (Figure 1). Notably, despite the switch to ARNI, there was no significant change in NT-proBNP levels, although a trend towards a decrease was observed (Figure 1, Table 2). A significant improvement in the NYHA functional class was observed following ARNI treatment (Table 2). Regarding laboratory parameters, no significant changes were noted for kidney function or in serum potassium levels.

### 3.3. ARNI Significantly Improved Hemodynamic Parameters of Both Systemic and Pulmonary Circulation

**Figure 2:**
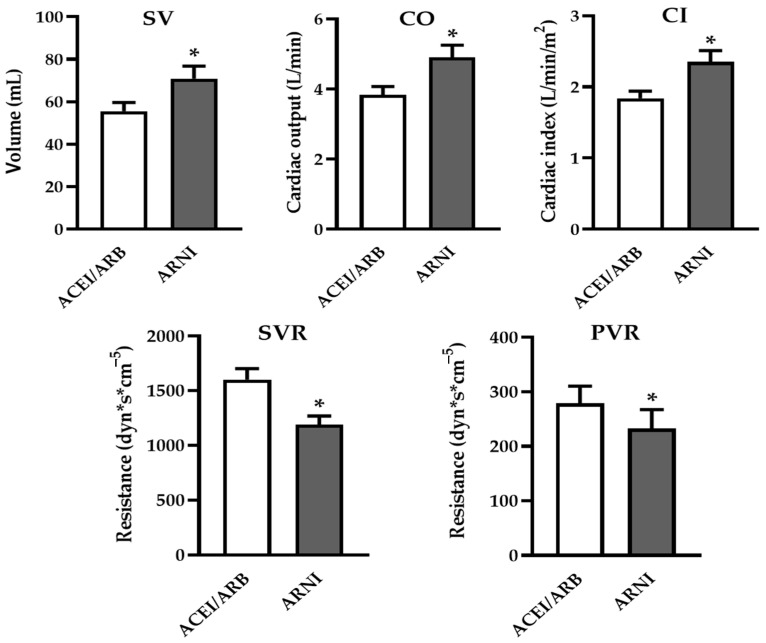
**Hemodynamic effects of ARNI**. After switching to ARNI, significant improvements in hemodynamic parameters were observed, and parameters relating to left ventricular systolic function improved significantly (stroke volume—SV; cardiac output—CO; and cardiac index—CI). Additionally, systemic vascular resistance (SVR) and pulmonary vascular resistance (PVR) also showed beneficial reductions. (Data are given as mean ± SEM (N = 13); * *p* < 0.05).

A significant increase was observed in cardiac output (CO: 4.9 ± 0.35 vs. 3.83 ± 0.24 L/min, *p* = 0.013), cardiac index (CI: 2.35 ± 0.16 vs. 1.84 ± 0.1 L/min/m^2^, *p* = 0.009), and stroke volume (SV: 70.9 ± 5.9 ml vs. 55.5 ± 4.12 ml, *p* = 0.012), indicating improved hemodynamic status following ARNI treatment. Furthermore, systemic vascular resistance (SVR: 1188 ± 79.8 vs. 1600 ± 100.5 dyn·s·cm^−5^, *p* = 0.004) significantly decreased (Figure 2, Table 2). However, no significant changes were detected in the systolic or diastolic blood pressure or heart rate. These findings suggest an overall improvement in systemic hemodynamics.

In the pulmonary circulation, systolic, mean, and diastolic pulmonary artery pressures (sPAP, mPAP, dPAP) did not change after switching to ARNI. However, a significant reduction in pulmonary vascular resistance (PVR: 232.5 ± 34.8 vs. 278.9 ± 31.7 dyn·s·cm^−5^, *p* = 0.04) (Figure 2, Table 2) was observed. This improvement in pulmonary circulation may enhance patients’ eligibility for HTX and may help to stabilize their status on the waiting list. No significant changes were noted in PCWP or CVP.

### 3.4. Clinical Outcomes

**Figure 3:**
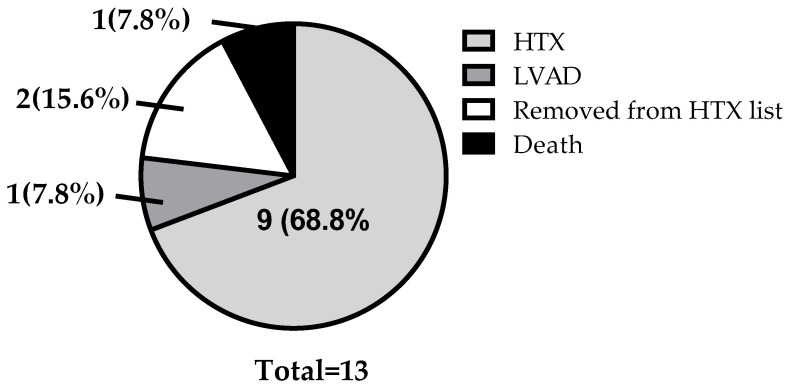
**Clinical outcomes in patients with AdHF after switching to ARNI**. (HTX, heart transplantation; LVAD, left ventricular assist device).

Following ARNI treatment, the long-term outcomes of AdHF patients were evaluated, including HTX, left ventricular assist device (LVAD) implantation, removal from the transplant list, and mortality. In the studied population, nine patients successfully underwent HTX, one patient received an LVAD, one patient died, and two patients were removed from the transplant list due to significant improvement in their functional status (Figure 3).

## 4. Discussion

The epidemiological and clinical characteristics of our study group align with international literature data. HF was predominantly observed in middle-aged and elderly patients, with a higher prevalence in men than in women [14,15]. Hypertension, type-2 diabetes, and coronary artery disease were the most prominent comorbidities and risk factors, while smoking history and obesity were also common [16]. While the efficacy and safety of ARNI in HF patients have been well-established in several clinical studies [5,6,17], limited data exist on the effects ARNI treatment on invasive hemodynamic parameters, making our study a valuable addition. According to the 2005 ESCAPE meta-analysis, routine use of Swan–Ganz (SG) catheterization to assess hemodynamic status in patients with HFrEF offers no advantage over non-invasive methods and carries the risk of central line-associated bloodstream infection (CLABSI), which has a high mortality rate [18,19]. For these reasons, SG catheterization has limited indications and is typically reserved for AdHF patients being evaluated for HTX or mechanical circulatory support. Patients on the transplant list are required to undergo the procedure every 3–6 months, depending on their PVR [4,20]. Overall, our repeated measurements among our patients on the transplant waiting list proved to be safe, and the revealed that ARNI significantly improves systemic circulatory parameters (SV, CO, CI, and SVR), likely due to its reverse remodeling effects [21,22,23].

The effects of ARNI on the systemic circulation have been demonstrated in several large-scale studies, but little data are available on its effects on the pulmonary circulation. A study by Zern et al., examining five end-stage HF patients, showed that ARNI reduced both PVR and mPAP [24]. In contrast, in our study, mPAP did not change significantly; however, a marked decrease in PVR in all but one patient could be observed irrespective of the presence or absence of postcapillary (secondary) pulmonary hypertension. The observed reduction in PVR suggests that ARNI may improve the suitability of AdHF patients for HTX. Another study, in 2021, investigated the effect of ARNI in rats with experimental pulmonary hypertension [25]. They found that ARNI administered in combination with bosentan had a significantly more beneficial effect on pulmonary vascular remodeling than either ARNI or bosentan alone. Understanding the effects of ARNI on pulmonary circulation is important because an irreversibly elevated PVR—i.e., still high after administering epoprostenol—is a relative contraindication for HTX [20,26].

In patients with AdHF, the CI is also important, with a value below 2.0 L/min/m^2^ being associated with poor prognosis. In those cases, urgent transplantation (high urgent (HU) listing) or mechanical circulatory support should be considered. Our data suggest that CI can be improved significantly by ARNI, which is in line with a study from 2021 in which 25 patients requiring intensive therapy (all CI < 2.2 L/per minute/m^2^) were treated with vasoactive (vasodilator, inotropic) therapy followed by ARNI. The findings indicate that following the administration of vasoactive agents, ARNI led to a further increase in the cardiac index. Our study reinforces these findings, demonstrating a significant increase in CI with ARNI, potentially improving prognosis and reducing the need for urgent interventions in this high-risk patient population. 

Target organ damage (e.g., CKD or hepatic dysfunction) is common in AdHF due to disease progression [27]. The transplant “golden window” is narrow; patients must be sick enough to require a new heart but stable enough to survive the procedure [28]. Therefore, timely recognition of therapeutic failure (e.g., via the I NEED HELP criteria) is crucial for prompt escalation and HTX consideration [29]. In this small cohort, a low incidence of cardiorenal syndrome was observed; only four patients had CKD, and none of them required renal replacement therapy.

The improvement in the NYHA functional class of our patients underscores the clinical relevance of the observed hemodynamic changes. While a trend towards decreasing NT-proBNP levels was noted, it is important to recognize that in AdHF, the impact of ARNI on NT-proBNP levels may be less pronounced than in patients with less-progressed stages of HF. 

Beyond the observed improvements in hemodynamic parameters, the long-term clinical effects of ARNI therapy warrant attention. In our study, we monitored patient outcomes following the initiation of ARNI treatment, and the results suggest significant clinical benefits. Among the 13 patients analyzed, 9 successfully underwent HTX, indicating that ARNI therapy might have contributed to the stabilization of their conditions and thus allowed them to remain eligible for transplantation. Additionally, only one patient required left ventricular assist device (LVAD) implantation, also highlighting ARNI therapy as an important therapeutic option in AdHF. Despite these positive outcomes, one patient experienced sudden cardiac death, underscoring the severity and life-threatening nature of end-stage heart failure, even in the presence of optimal medical therapy. Notably, two patients demonstrated such substantial clinical improvements that they could be removed from the transplant list. These findings further support the potential role of ARNI therapy in enhancing cardiac function and improving overall clinical status, even in patients with AdHF.

Overall, our results imply that ARNI therapy not only improves hemodynamic parameters but has a favorable impact on patients’ long-term outcomes. The high rate of transplantations and removals from the transplant list suggest that ARNI treatment may be a valuable tool in managing patients with AdHF, potentially improving their survival and quality of life.

## 5. Study Limitations

Our study has several limitations. Its retrospective design and small sample size limit the generalizability and statistical power of the findings. Nevertheless, despite the small number of patients involved, the magnitudes of the ARNI effects were remarkable, being suggestive of a sufficient statistical power to detect significant differences in most investigated parameters. We also anticipate that the lack of a control group makes it difficult to attribute improvements solely to ARNI as other factors, such as disease progression, may have influenced hemodynamic changes. Additionally, while significant hemodynamic improvements were observed, NT-proBNP levels did not change significantly, possibly due to the advanced HF stage or the limited sample size.

Despite these limitations, our study offers valuable insights into the hemodynamic effects of ARNI in AdHF patients awaiting HTX. The observed improvements in systemic and pulmonary circulation, along with a trend towards better clinical outcomes, suggest that ARNI may play a role in stabilizing this high-risk population.

## 6. Conclusions

ARNI therapy enhances hemodynamic parameters in both the pulmonary and systemic circulation in patients with AdHF. Our findings indicate that ARNI plays a pivotal role in the stabilization and clinical improvement of this high-risk patient population, who otherwise face a poor prognosis. While its effect on NT-proBNP levels may be less pronounced in advanced HF stages, the hemodynamic benefits of ARNI therapy remain clinically significant. These results underscore the need for further research to evaluate the impact of ARNI on pulmonary hemodynamics in a larger HF cohort.

## Figures and Tables

**Table 1:** Baseline characteristics of AdHF patients before initiation of ARNI.

Parameter	Study Population (N = 13)
Age, n (%)	56.4 ± 2.49
Female, n (%)	3 (23)
Male, n (%)	10 (77)
BMI, (kg/m^2^)	27.9 ± 2.82
Time on ARNI therapy, (month, IQR)	6.8 (4.9–16.1)
Etiology, n (%)	
Ischemic	6 (46.2)
Non-ischemic	7 (53.8)
NYHA class, n (%)	
III	11 (84.5)
IV	2 (15.5)
GDMT, n (%)	
ACEI/ARB	13 (100)
BB	13 (100)
MRA	13 (100)
SGLT2 inhibitor	4 (31)
≥50% of target GDMT, n (%)	
ACEI/ARB	13 (100)
BB	12 (92.7)
MRA	10 (76.9)
SGLT2 inhibitor	4 (31)
Device therapy, n (%)	
ICD	12 (92.3)
CRT-D	1 (7.7)
Comorbidities, risk factors n (%)	
Hypertension	7 (54)
Diabetes mellitus (type-2)	4 (31)
Previous MI	4 (31)
Atrial fibrillation	6 (46)
CKD	4 (31)
COPD	3 (23)
Hyperlipidemia	7 (54)
Previous smoker	5 (39)
Previous alcohol consumption	2 (15.5)

Data are given as mean ± SEM or median and interquartile range. Abbreviations: ARNI, angiotensin receptor blocker neprilysin inhibitor; NYHA, New York Heart Association, GDMT, guideline directed medical therapy; ACEI, angiotensin convertase enzyme inhibitor; ARB, angiotensin receptor blocker; BB, beta-blocker; MRA, mineralocorticoid receptor antagonist; SGLT2 inhibitor, sodium–glucose cotransporter-2 inhibitor; ICD, implantable cardioverter defibrillator; CRT-D, cardiac resynchronization therapy; MI, myocardial infarction; CKD, chronic kidney disease; COPD, chronic obstructive pulmonary disease.

**Table 2:** Changes in NYHA class, medical therapy, laboratory data, echocardiographic, and hemodynamic parameters in AdHF patients following ARNI initiation.

Parameter	ACEI/ARB Therapy (N = 13)	ARNI Therapy (N = 13)	*p*-Value
**NYHA class, n (%)**			
**I**	**0 (0)**	**1 (7.8)**	
**II**	**0 (0)**	**6 (46.1)**	**<0.001**
**III**	**11 (84.5)**	**6 (46.1)**	
**IV**	**2 (15.5)**	**0**	
GDMT, n (%)			
ACEI	13 (100)	0 (0)	
ARNI	0 (0)	13 (100)	n.a.
BB	13 (100)	13 (100)	
MRA	13 (100)	13 (100)	
SGLT2 inhibitor	4 (31)	4 (31)	
≥50% of target GDMT, n (%)			
ACEI	13 (100)	0 (0)	
ARNI	0 (0)	10 (76.9)	n.a.
BB	12 (92.7)	12 (92.7)	
MRA	10 (76.9)	12 (92.7)	
SGLT2 inhibitor	4 (31)	4 (31)	
Target dose of ARNI, n (%)			
<50%	n.a.	10 (76.9)	n.a.
≥50%	n.a.	3 (23.1)	n.a.
Laboratory parameters			
NT-proBNP (pg/mL)	2847 (1444–4977)	2055 (1313–2754)	0.092
eGFR (mL/min/1.73 m^2^)	70.8 ± 5.18	67.9 ± 5.69	0.433
Serum creatinine (µmol/L)	97.9 ± 7.32	103.1 ± 8.53	0.279
BUN (mmol/L)	8.6 ± 1.07	9.5 ± 1.12	0.223
Potassium (mmol/L)	4.39 ± 0.13	4.5 ± 0.11	0.533
**Echocardiography**			
LVEF (%)	**23.65 ± 1.02**	**27.27 ± 1.04**	**0.016**
EDD (mm)	75 ± 3.04	77.1 ± 2.61	n.s.
**Hemodynamics**			
sSBP (mmHg)	116 (98–124.5)	106 (101–116)	0.197
dSBP (mmHg)	67.9 ± 2.84	62.8 ± 1.85	0.193
mSBP (mmHg)	85 (75–86.5)	79 (72.5–84)	0.260
sPAP (mmHg)	46 ± 3.42	43.8 ± 3.52	0.421
dPAP (mmHg)	22 ± 1.4	19.6 ± 1.6	0.118
mPAP (mmHg)	32 (25.5–38.5)	27.92 (17.5–35)	0.168
**SVR (dyn·s·cm^−5^)**	**1600 ± 100.5**	**1188 ± 79.8**	**0.004**
**PVR (dyn·s·cm^−5^)**	**278.9 ± 31.7**	**232.5 ± 34.8**	**0.04**
**SV (mL)**	**55.5 ± 4.12**	**70.9 ± 5.9**	**0.013**
**CO (L/min)**	**3.83 ± 0.24**	**4.9 ± 0.35**	**0.013**
**CI (L/min/m^2^)**	**1.84 ± 0.1**	**2.35 ± 0.16**	**0.009**
HR (1/min)	70 ± 1.8	71 ± 2.71	0.593
CVP (mmHg)	8.31 ± 1.62	6.62 ± 1.16	0.321

Data are given as means ± SEM or medians and interquartile ranges. The significant results are highlighted in bold in the table. Abbreviations: LVEF, left ventricular ejection fraction; EDD, end diastolic diameter; ARNI, angiotensin receptor neprilysin inhibitor; NYHA, New York Heart Association, GDMT, guideline directed medical therapy; ACEI, angiotensin convertase enzyme inhibitor; ARB, angiotensin receptor blocker; BB, beta-blocker; MRA, mineralocorticoid receptor antagonist; SGLT2 inhibitor, sodium–glucose linked transporter 2 inhibitor; sSBP, systolic systemic blood pressure; dSBP, diastolic blood pressure; mSBP, mean systemic blood pressure; sPAP, systolic pulmonary arterial pressure; dPAP, diastolic pulmonary arterial pressure; mPAP, mean pulmonary arterial pressure; SVR, systemic vascular resistance; PVR, pulmonary vascular resistance; CO, cardiac output; CI, cardiac index; HR, heart rate; CVP, central venous pressure; NT-proBNP, N-terminal pro-brain natriuretic peptide; eGFR, estimated glomerular filtration rate; BUN, blood urea nitrogen; n.a.: not applicable or not available.

## Data Availability

The data analyzed and presented in this study are available from the corresponding author on request.
